# Interplay between vitamin D status, vitamin D receptor gene variants and preeclampsia risk in Ghanaian women: A case-control study

**DOI:** 10.1371/journal.pone.0303778

**Published:** 2024-05-30

**Authors:** Linda Ahenkorah Fondjo, Jonah Buah Mensah, Enoch Ofori Awuah, Samuel Asamoah Sakyi

**Affiliations:** Department of Molecular Medicine, KSMD, KNUST, Ghana; University of Cape Coast, GHANA

## Abstract

**Background and aim:**

Preeclampsia (PE) is characterized by hypertension and proteinuria mostly after 20 weeks of gestation. It affects 2–8% of pregnancies worldwide, with detrimental consequences for both mother and foetus. Evidence, suggests that genetic factors, including vitamin D receptor (VDR) gene polymorphisms, could contribute to PE complexity. However, their role in the Ghanaian population remains underexplored. We assessed the interplay between Vitamin D, VDR gene variants and preeclampsia risk in Ghanaian women.

**Methods:**

This unmatched case-control study was conducted at Kumasi South Hospital, Ghana, from June to November 2022. A total of 162 participants consisting of 62 PE cases and 100 normotensive controls were enrolled. Clinical and obstetric data were collected. Blood samples were also collected for DNA extraction and vitamin D assay. Genotyping of VDR Fok1 and Bsm1 gene variants was performed using Polymerase Chain Reaction (PCR) and Polymerase Chain Reaction—Restriction Fragment Length Polymorphism (PCR-RFLP) analysis whereas Vitamin D levels were estimated using sandwich ELISA. Statistical analyses were computed with SPSS version 25 and GraphPad prism version 8.0. A p-value of < 0.05 was considered statistically significant.

**Results:**

Vitamin D concentration were significantly lower in the PE group (*p* < 0.0001). Vitamin D deficiency (aOR = 3.311, 95% CI: 1.584–6.921, p = 0.0010) was significantly associated with a three-fold increase in preeclampsia risk, whilst VDR gene variants, particularly the "bb" genotype (cOR = 0.227, 95% CI: 0.055–0.944, p = 0.0410) was associated with reduced risk of PE. There was no association between the distribution of Fok1 genotypes and PE.

**Conclusion:**

This study highlights a significant association between vitamin D deficiency and an increased risk of PE among Ghanaian women. However, the VDR gene variant, "bb", genotype, for Bsm1 reduces the risk of PE.

## Background

Preeclampsia (PE) is a pregnancy-specific complication, symptomized by hypertension and proteinuria usually occurring 20 weeks into gestation. Preeclampsia significantly contributes to maternal mortality [1], particularly in low and middle-income countries like Ghana where recent studies indicate hypertensive disorders of pregnancy have surpassed haemorrhage as the leading cause of maternal death [2]. With a global prevalence of 2–8%, untimely diagnosis of preeclampsia can lead to life threatening conditions such as eclampsia, disseminated intravascular coagulation, and HELLP syndrome as well as intrauterine growth restriction of the foetus [1].

The pathogenesis of PE involves complex biological processes, encompassing oxidative stress, maternal inflammation, improper placental perfusion, and impaired spiral artery remodelling [3]. The precise aetiology of preeclampsia remains incompletely understood, making early identification and diagnosis of individuals at risk of developing the condition difficult thereby rendering management burdensome [3, 4]. This obscure nature of preeclampsia has necessitated many studies into assessing the factors that influence the development of preeclampsia. The emergence of vitamin D deficiency as a factor that contributes to the risk of PE has also garnered increasing attention in recent years [1].

Evidence from some studies have implicated vitamin D deficiency as a risk factor influencing the development of preeclampsia [3]. Vitamin D is known to exert its protective effects against preeclampsia through its involvement in anti-inflammatory, immune-modulatory, blood pressure regulation processes as well as antioxidant mechanisms [3, 5]. The active form of vitamin D, 1,25(OH)_2_D, has been known to exert its functions through vitamin D receptors (VDR) expressed in various organs, including the placenta [6].

The VDR gene, located on chromosome 12, is capable of regulating a significant portion of the human genome, including cell proliferation, immune function, blood pressure, and cellular differentiation [6, 7]. Variability within the VDR gene, is known to influence its protein levels and their stability, potentially contributing to the risk of developing preeclampsia. Two VDR variants; rs228570 (Fok1) and rs1544410 (Bsm1) have been associated with the increased susceptibility to preeclampsia and hypertension among Italians, Chinese and Iranians [8–10].

Although, some studies have drawn associations between vitamin D deficiency and preeclampsia risk, other research findings have been inconsistent. Some of these studies have suggested an association between low maternal vitamin D levels and the development of PE, while others have found no such evidence [3, 11]. In Ghana, PE poses a significant threat to maternal and foetal health, evident by current prevalence of 6.8% [12]. We have earlier reported low maternal vitamin D status in both preeclamptic and normal pregnancies [13], warranting further evaluation of the influence of vitamin D deficiency and VDR gene variants on women experiencing PE. This study therefore investigated the interplay between vitamin D status and VDR gene variants in association with preeclampsia risk in the Ghanaian population.

## Materials and methods

### Study design and study site

This unmatched case-control study was conducted using a cohort of pregnant women diagnosed with preeclampsia as cases; and normotensive pregnant women as controls. The study participants were recruited from the Kumasi South Hospital, Atonsu, in Kumasi, Ashanti Region of Ghana, during their regular antenatal visits, between June 2022 to November 2022. The Kumasi South Hospital is located in Kumasi, the 2^nd^ largest city in Ghana that is on latitude N06°41.37 and longitude W001°36.65. It is located in the southern central part of Ghana in the Ashanti Region and has a population of about 3.5 million people according to Ghana Statistical Services in 2021. The hospital, serves as the second leading referral centre in the Ashanti region with a bed capacity of about 132 making it a suitable place for the study.

### Study population

The study recruited Ghanaian women aged 18-45years with singleton pregnancies who developed preeclampsia after the 20th week of gestation. Diagnosis of preeclampsia was made by a qualified obstetrician as part of standard of care following the ISSHP criteria. The ISSHP guidelines for the diagnosis of preeclampsia defines preeclampsia as the new onset of high blood pressure (greater than 140/90 mmHg) and one or more of the following; proteinuria (≥1+ reading on a dipstick) after the 20th week of gestation, maternal organ and uteroplacental dysfunction in individuals who were previously normotensive [14]. The selected control participants were pregnant women who did not have preeclampsia or other pregnancy complications. All participants in the study were of Ghanaian nationality and provided informed consent.

The study excluded pregnant women below 18 years and above 45 years of age, as well as those with twin gestation. Additionally, individuals diagnosed with chronic or gestational hypertension were excluded. Pregnant women with co-morbid medical conditions, such as diabetes, cardiovascular disorders, antepartum haemorrhage, and various endocrine conditions (hyperthyroidism, hypothyroidism, gestational diabetes, types 1 and 2 diabetes), were also excluded from the study.

### Sample size calculation and sampling technique

The minimum sample size for cases was determined using the Kelsey’s formula, considering odds ratio (OR) of 2.0 and 7.03% assumed exposure among controls (prevalence) of pre-eclampsia in Ghana [15] at 95% confidence level and 80% power. In total 162 patients were recruited for this study. This consisted of 100 normotensive controls and 62 preeclamptic cases. A purposive sampling technique was employed.

### Ethical considerations

Ethical approval for this study was obtained from the Kumasi South Hospital Ethics Review committee and the Committee on Human Research, Publication and Ethics (CHRPE/AP/243/22) of the Kwame Nkrumah University of Science and Technology. Prospective study participants volunteered to participate in the study after a thorough informed consent process. The consent document outlined the purpose of the study including the procedures and risks involved in participating in the study. The consent process involved explaining the objectives of the study, the procedures, risks, benefits, and voluntary nature, permitting participants to make an informed decision to partake in the study. All participants provided a written informed consent to partake in the study in the form of signature or fingerprint before the start of the study. Strict measures ensured confidentiality and privacy, data obtained was anonymized and securely stored accessible only to researchers involved in this study. This study was conducted in accordance with the guidelines of the Helsinki Declaration.

### Sample and data collection

A well-structured questionnaire was administered to all study participants to obtain relevant clinical data. For each participant, 8 mL of blood was collected, which was subsequently evenly divided into two separate tubes; an Ethylenediaminetetraacetic Acid (EDTA) and a gel separator tubes, containing 4 mL of blood respectively. Samples collected into gel separator tubes were centrifuged at 2000 g for 5 minutes, and separated serum were collected into sterile cryotubes and stored at -80°C until assayed for vitamin D (25(OH)D). Blood samples collected into EDTA tubes were stored at -20°C until required for DNA extraction.

### DNA extraction for Fok1 and Bsm1 VDR variants

Genomic DNA was extracted from blood samples using the double salt precipitation method. The quality and purity of the extracted DNA were assessed using a UV-Vis spectrophotometer (Thermo Scientific™ GENESYS™ 180 UV-Visible Spectrophotometer, USA) to ensure good quality DNA samples were used for subsequent procedures. Purity of DNA was acceptable when the 260/280 nm ratio lay between 1.8 and 2.00 using the NanoDrop™. The extracted DNA was used for the analyses of Fok1 and Bsm1 VDR variants using polymerase chain reaction (PCR) and the restriction fragment length polymorphism (RFLP).

### DNA amplification

The DNA amplification was carried out using polymerase chain reaction (PCR) in a thermal cycler (Applied Biosystems 2720, UK) for rs228570 (Fok1) and rs1544410 (Bsm1) separately. The primer sequences (New England Biolabs, USA) for both variants are presented in the **Table 1.**

**Table 1:** Primer sequences used for the PCR reaction.

rs Number	Name	Sequence
rs 2228570	Fok1 F	AGCTGGCCCTGGCACTGACTCTGCTCT
	Fok1 R	ATGGAAACACCTTGCTTCTTCTCCCTC
rs 1544410	Bsm1 F	AACCAGCGGGAAGAGGTCAAGGG
	Bsm1 R	CAACCAAGACTACAAGTACCGCGTCAGTGA

F = forward; R = reverse

The total PCR reaction volume was 25μl, which comprised 10μM solutions of each individual primer, 2X PCR master mix and nuclease-free water (New England Biolabs, USA). The volumes of each of the components contained in the 25 μl PCR reaction were; 7.5 μl ddH2O, 12.5 μl 2X PCR master mix, 0.5 μl forward primer, 0.5 μl reverse primer and 4 μl DNA template.

The cycling conditions for Fok1 amplification were an initial holding denaturation for 2 min at 94°C, followed by 30 cycles of denaturation at 94°C for 30 s, annealing at 62°C for 30 s, extension at 68°C for 30 s and a final extension at 68°C for 5 min. For Bsm1, initial holding denaturation for 2 min at 94°C, followed by 30 cycles of denaturation at 94°C for 30 s, annealing at 61°C for 1 min, extension at 68°C for 1 min and a final extension at 68°C for 5min.

### SNP genotyping

For the SNP detection of Fok1 and Bsm1, the amplicons from each amplification process were utilized for enzyme digestion using Fok1and Bsm1 restriction enzymes (New England Biolabs, USA) respectively. The procedures were carried out for both polymorphisms as described by Rezende *et*.,*al* [16], with band sizes for Fok1 as follows; two bands at 272 bp and 198 bp, which represented the “Ff” genotype. A single band at 272 bp indicated the presence of the wild type, which is the “FF” genotype, while the “ff” genotype corresponded to 198 bp.

Bsm1 restriction enzyme digestion had band sizes of 825 bp, 650 bp, and 175 bp, which corresponded to “Bb” genotype, a single band at 825 bp indicating the absence of the SNP, and denoted the “BB” genotype, while the presence of only 175 bp band denoted the “bb” genotype.

### Agarose gel electrophoresis

**Fig 1:**
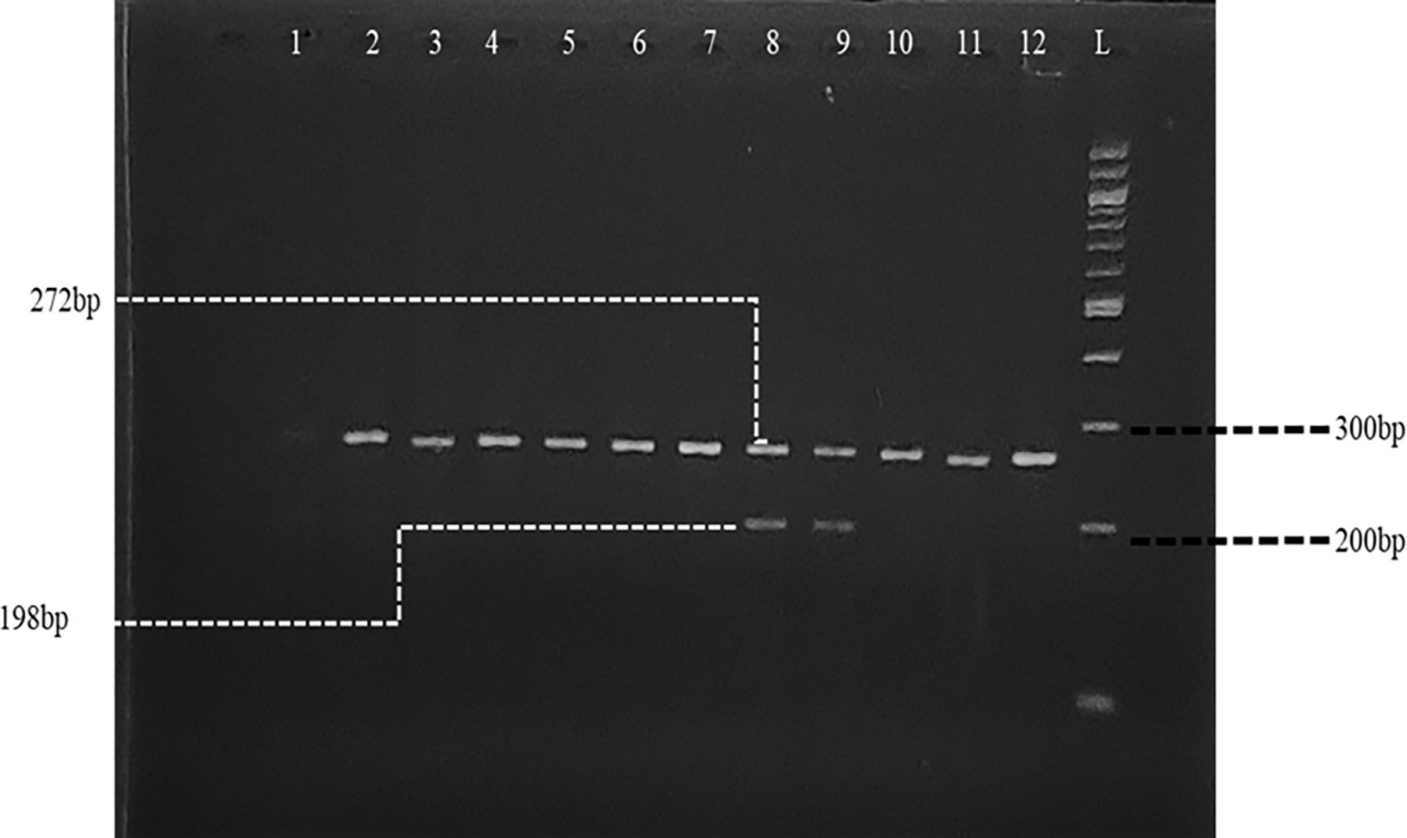
Gel image post PCR-RFLP reaction for Fok1. Gel image showing the restriction enzyme digestion for Fok1, corresponding lane numbers and their genotype descriptions 1–7 & 10–12 = “FF”, 8&9 = “Ff”.

**Fig 2:**
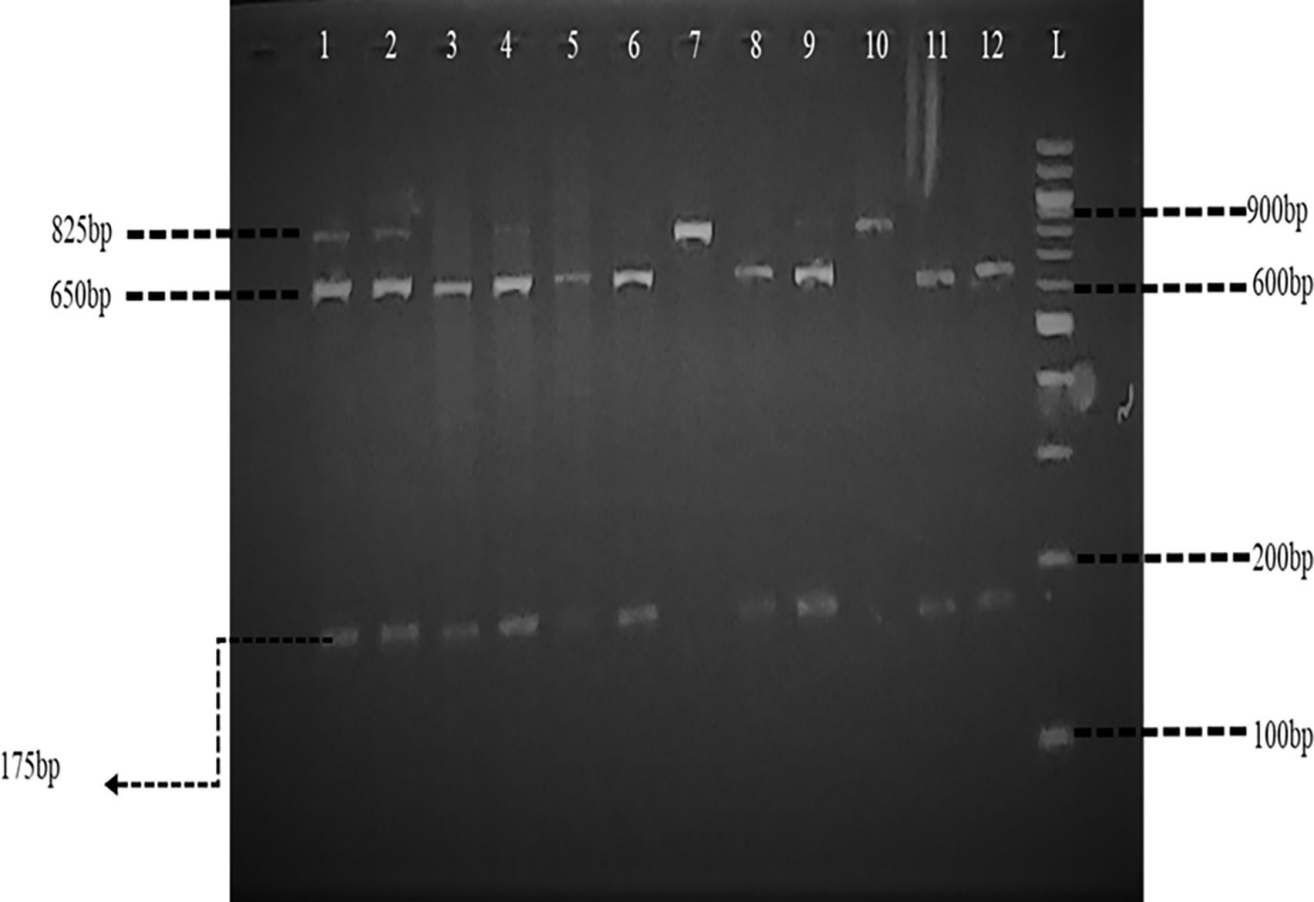
Gel image post PCR-RFLP reaction for Bsm1. Gel image showing the restriction enzyme digestion for Bsm1, corresponding lane numbers and their genotype descriptions are 7 & 10 = “BB”, 1, 2 & 4 = “Bb” and 3, 5, 6, 8, 9, 11, & 12 = “bb”.

Following the restriction endonuclease digestion, DNA fragments were separated using the agarose gel electrophoresis technique at 3% concentration of agarose and the subsequent visualization of bands using a UV transilluminator (Cleaver Scientific, UK). The gel was prepared with ethidium bromide to enhance visualization under UV **(Figs 1 and 2)**. The electrophoresis unit (Cleaver Scientific, UK) was filled with Tris–Borate–EDTA (TBE) buffer, 10 μl of 100 bp molecular ladder (Meridian Bioscience, USA) was loaded and 10 μl of DNA samples were also loaded.

The fragmentation of post restriction endonuclease digestion for Fokl SNP resulted in bands of sizes that depict a participant’s genotype **(Fig 1)**. The presence of only the 272 bp denotes homozygous “FF” genotype, 272 bp and 198 bp denote heterozygous “Ff”, and the presence of only the 198 bp band correspond to homozygous recessive “ff” genotype.

Fragmented Bsml digested by the Bsml restriction enzyme resulted in band sizes that depict the genotypes of participants as homozygous “BB” (825 bp), homozygous recessive “bb” (650 bp and 175 bp) and heterozygous “Bb” (825 bp, 650 bp and 175 bp) **(Fig 2)**.

### Vitamin D estimation

Stored serum were allowed to thaw at room temperature for the measurement of 25(OH)D using a sandwich enzyme linked-immunosorbent assay (ELISA) kits, following the manufacturer’s protocol. Absorbances were read at 450nm using a microplate reader (RT-2100C microplate reader, Rayto). The concentration of serum vitamin D in test samples were estimated from the standard curve plotted using the different standards with known concentrations. For the estimation of vitamin D, 14 samples were lost during storage, thus 148 stored samples were used for the estimation of vitamin D. Vitamin D concentrations < 20 ng/mL were considered as vitamin D deficiency.

### Statistical analyses

Statistical analyses were computed using Statistical Package for Social Sciences (SPSS) Version 26.0 (Chicago IL, USA) and GraphPad prism version 8.0 (GraphPad software, San Diego California USA, www.graphpad.com). The prevalence of Fok1 and Bsm1 gene variants within the study population was determined by calculating the proportion of each gene variant’s occurrence relative to the total number of participants. Categorical variables were presented as frequencies and percentages. The categorical variables included parity, gravidity, weight category, ethnicity, history of preeclampsia and hypertension. The associations with disease outcomes were evaluated using either the Chi-square test or Fisher’s exact test when applicable. All parametric continuous variables were presented as (mean ± SD) based on normality test with Kolmogorov–Smirnov test and differences were evaluated using the independent sample t-test. Chi-square was used to determine the association between Fok1 and Bsm1 SNPs with vitamin D status, and their association with preeclampsia. The extent of this association was determined through Binary logistic regression testing, and the results were presented as crude odds ratios (cOR) or adjusted odds ratios (aOR). These ratios provided information on the degree of association with risk of preeclampsia or protective effect. A p-value of < 0.05 was considered statistically significant.

## Results

### Baseline characteristics of study participants

The baseline characteristics of the study participants are shown in **Tables 2 and 3**. This study included a total of 162 participants, the age range of study participants was between 18 and 43 years. Notably, the PE participants had significantly higher BMI (Body Mass Index) (p = 0.0160) and weight (p = 0.0010).

**Table 2:** Clinical and anthropometric characteristics of study participants.

Variables	Normotensives n = 100(Mean ±SD)	Preeclampsia n = 62(Mean ±SD)	p-value (p<0.05)
Age (years)	28.73 ± 6.252	30.15 ± 6.277	0.1130
Gestational age (weeks)	31.38 ± 4.863	32.26 ± 4.581	0.3950
Weight (kg)	72.541 ± 14.0910	77.500 ± 10.1186	**0.0010**
Systolic (mmHg)	111.44 ± 11.634	156.65 ± 14.487	**<0.0001**
Diastolic (mmHg)	68.90 ± 9.341	103.63 ± 7.893	**<0.0001**
Height (m)	1.59 ± 0.0674	1.60 ± 0.0729	0.1170
BMI	28.84 ± 5.4861	30.44 ± 5.1826	**0.0160**

BMI = Body Mass Index, p-values computed by independent sample t—test, statistical significance at p < 0.05

**Table 3:** Socio-demographic and obstetric characteristics of study participants.

Variables	Normotensives n = 100 (%)	Preeclampsia n = 62 (%)	p-value
BMI Category			0.0370
Normal	26(26)	6(9.7)	
Overweight	41(41)	29(46.8)	
Obese	33(33)	27(43.5)	
**First pregnancy**			0.1000
No	91(91)	51(82.3)	
Yes	9(9)	11(17.7)	
**Gravidity**			0.3490
Primigravida	10(10)	11(17.7)	
Secundigravida	23(23)	12(19.4)	
Multigravida	67(67)	39(62.9)	
**Parity**			0.6390
Nullipara	19(19)	15(24.2)	
Primipara	26(26)	13(21)	
Multipara	55(55)	34(54.8)	
**History of preeclampsia**			<0.0001*
No	97(97)	41(66.1)	
Yes	3(3)	21(33.9)	
**Family History of Hypertension**			0.1290
No	94(94)	54(87.1)	
Yes	6(6)	8(12.9)	
**Occupation**			**0.0010**
Unemployed	21(21)	6(9.7)	
Self-employed	56(56)	24(38.7)	
Employed	23(23)	32(51.6)	
**Ethnicity**			
Akan	71(71)	53(42.7)	0.1510*
Ewe	7(7)	2(22.2)	
Ga	4(4)	0(0)	
Northerner	18(18)	7(28)	
**Marital Status**			0.5370*
Divorced	2(2)	3(4.8)	
Married	58(58)	37(59.7)	
Single	40(40)	22(35.5)	
**Contraceptive Usage**			0.7180
No	72(72)	43(69.4)	
Yes	28(28)	19(30.6)	
**Alcohol intake**			0.7430*
No	93(93)	59(95.2)	
Yes	7(7)	3(4.8)	

BMI: Body mass index, Chi-square and Fisher’s (*) exact test were used where appropriate, p-value is statistically significant at p<0.05

A higher proportion of PE women (33.9%) had a previous history of preeclampsia, whereas only 3% of normotensive pregnant women reported history of PE with the difference being statistically significant (p = <0.0001).

### Vitamin D deficiency and preeclampsia risk

Table 4 shows the concentrations of 25(OH)D was significantly lower in the PE group compared to the normotensive pregnant group (*p* <0.0001). The overall prevalence of vitamin D deficiency in the 148 study participants was 54.7%. Of the women with preeclampsia, 70.9% had vitamin D deficiency, while 45.2% of normotensive pregnant women had vitamin D deficiency. Logistics regression analysis showed that participants with vitamin D deficiency had approximately a 3-fold increase in the odds of developing preeclampsia (cOR = 2.960, 95% CI: 1.454–6.026, *p = 0*.*0030*). After adjusting for age and BMI, the odds increased slightly (aOR = 3.311, 95% CI: 1.584–6.921, *p* = 0.0010).

**Table 4:** Association between Vitamin D and the risk of preeclampsia.

Variable	Normotensive (n = 93)	Preeclampsia (n = 55)	p-value	cOR (95% CI)	p-value	aOR (95% CI)	p-value
**Vitamin D (mean ± SD)**	21.37 ± 5.5030	17.34 ± 4.5571	<0.0001^a^	0.807 (0.729–0.894)	<0.0001^c^	0.800 (0.719–0.890	<0.0001^c^
**Vitamin D Status**	**n = 93(%)**	**n = 55(%)**		1.00	**-**	1.00	**-**
Deficiency (<20ng/ml)	42 (45.2)	39 (70.9)	0.002^b^	2.960 (1.454–6.026)	0.0030^c^	3.311 (1.584–6.921)	0.0010^c^
Sufficiency (>20ng/ml)	51 (54.8)	16 (29.1)		1.00	**-**	1.00	**-**

^a^: p-value computed by independent sample t-test

^b^: p-value computed by Chi-square Test

^c^: p-value computed by logistics regression model

Frequency-n, Crude odds ratio (cOR), Adjusted odd ratio (aOR), CI (95% confidence interval), adjusted for age and BMI, p-value statistically significant at p < 0.05

**Fig 3:**
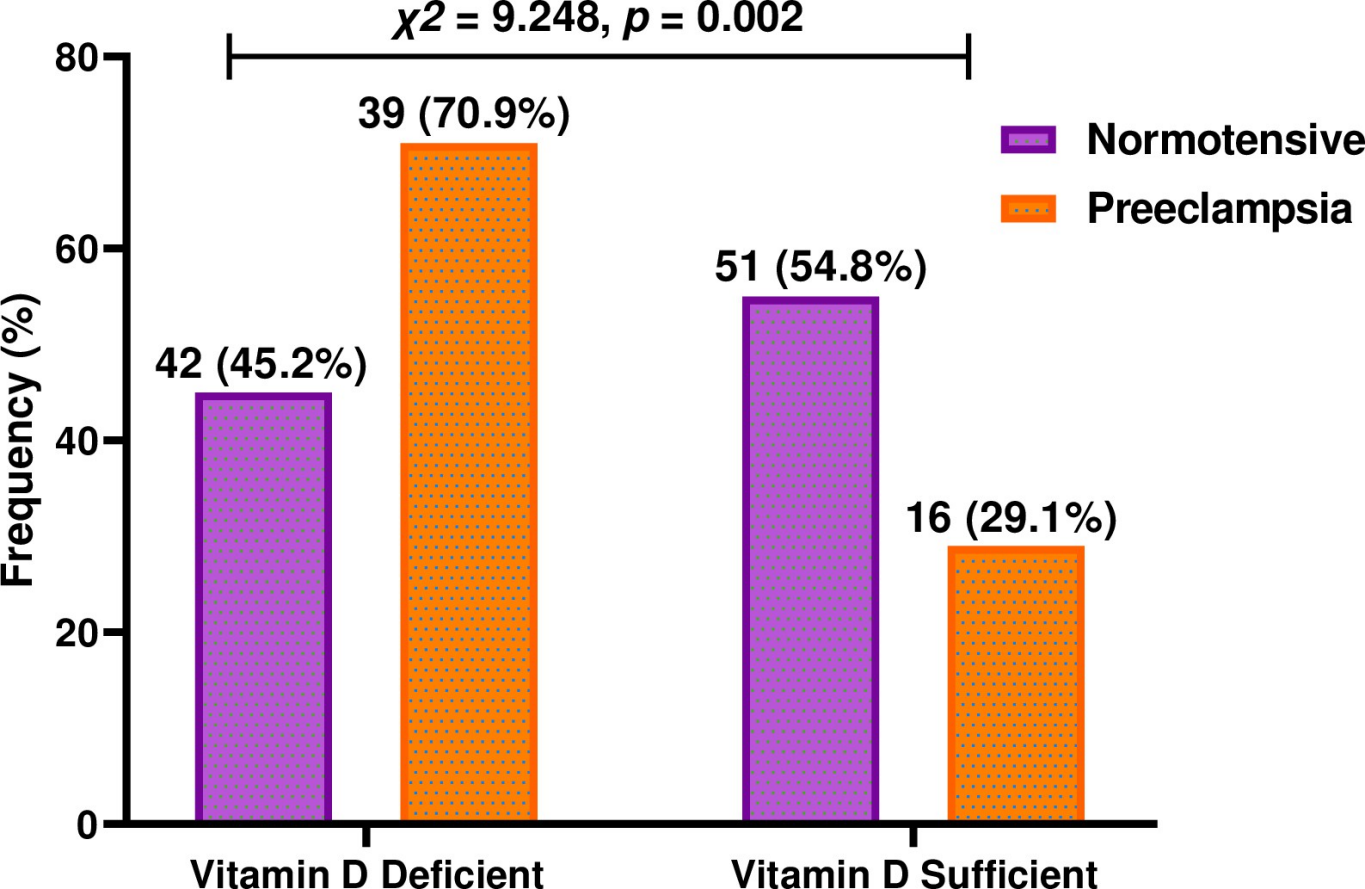
Vitamin D status among the study participants.

**Fig 3** shows the vitamin D status of the two study groups. There was a significant difference in vitamin D levels between the two groups (*p* = 0.002). With the normotensive group being more vitamin D sufficient and the preeclampsia group being more vitamin D deficient.

### VDR gene variants and the risk of preeclampsia

Table 5 shows the distribution of Fok1 and Bsm1 genotype variants among the NP and PE groups. While Fok1 genotypes did not show a significant difference, there was a significant difference in the distribution of Bsm1 genotypes between the groups (*p* = 0.001). In the dominant model, no significant difference was observed for both Fok1, as well as Bsm1 (*p* = 0.809) and (*p* = 0.635) respectively. However, the crude odds ratio analysis in Table 6 revealed that possessing the “bb” genotype (homozygous for the recessive Bsm1 allele) reduced the odds of developing preeclampsia (cOR = 0.227, 95% CI: 0.055–0.944, *p* = 0.0410).

**Table 5:** Genotype distribution and association of VDR gene variants among study participants.

Variables	Normotensive n = 100(%)	Preeclampsia n = 62(%)	(χ2, df) p-value
**Fok1 Genotype**			(0.061, 2) 0.9700
FF	61(61)	39(62.9)	
Ff	24(24)	14(22.6)	
ff	15(15)	9(14.5)	
***Dominant***			(0.59, 1) 0.8090
FF	61(61)	39(62.9)	
Ff + ff	39(39)	23(37.1)	
**Bsm1 Genotype**			(13.875, 2) **0.0010**
BB	9(9)	7(11.3)	
Bb	55(55)	49(79)	
bb	36(36)	6(14.3)	
***Dominant***			(0.226, 1) 0.6350
BB	9(9)	7(11.3)	
Bb + bb	91(91)	55(88.7)	

χ2 –chi-square test, df- degrees of freedom, p-value <0.05

**Table 6:** Genotype distribution and the risk of preeclampsia among study participants.

VDR Gene Polymorphisms	Normotensive N = 100(%)	Preeclampsia N = 62(%)	OR (95% CI)	p-value
**Fok1**				
FF	61(61)	39(62.9)	1.00	
Ff	24(24)	14(22.6)	0.990 (0.440–2.229)	0.9810
ff	15(15)	9(14.5)	1.094 (0.411–2.229)	0.8570
***Dominant***				
FF	61(61)		1.00	
Ff+ff	39(39)		0.935 (0.485–1.803)	0.8410
**Bsm1**				
BB	9(9)	7(11.3)	1.00	
Bb	55(55)	49(79)	1.219 (0.371–4.004)	0.7450
bb	36(36)	6(14.3)	0.227 (0.055–0.944)	**0.0410**
***Dominant***				
BB	9(9)	7(11.3)	1.00	
Bb+bb	91(91)	55(88.7)	0.785 (0.275–2.237)	0.6500

OR–crude odds ratio, CI– 95% confidence interval. statistical significance of p-value at <0.05

**Fig 4:**
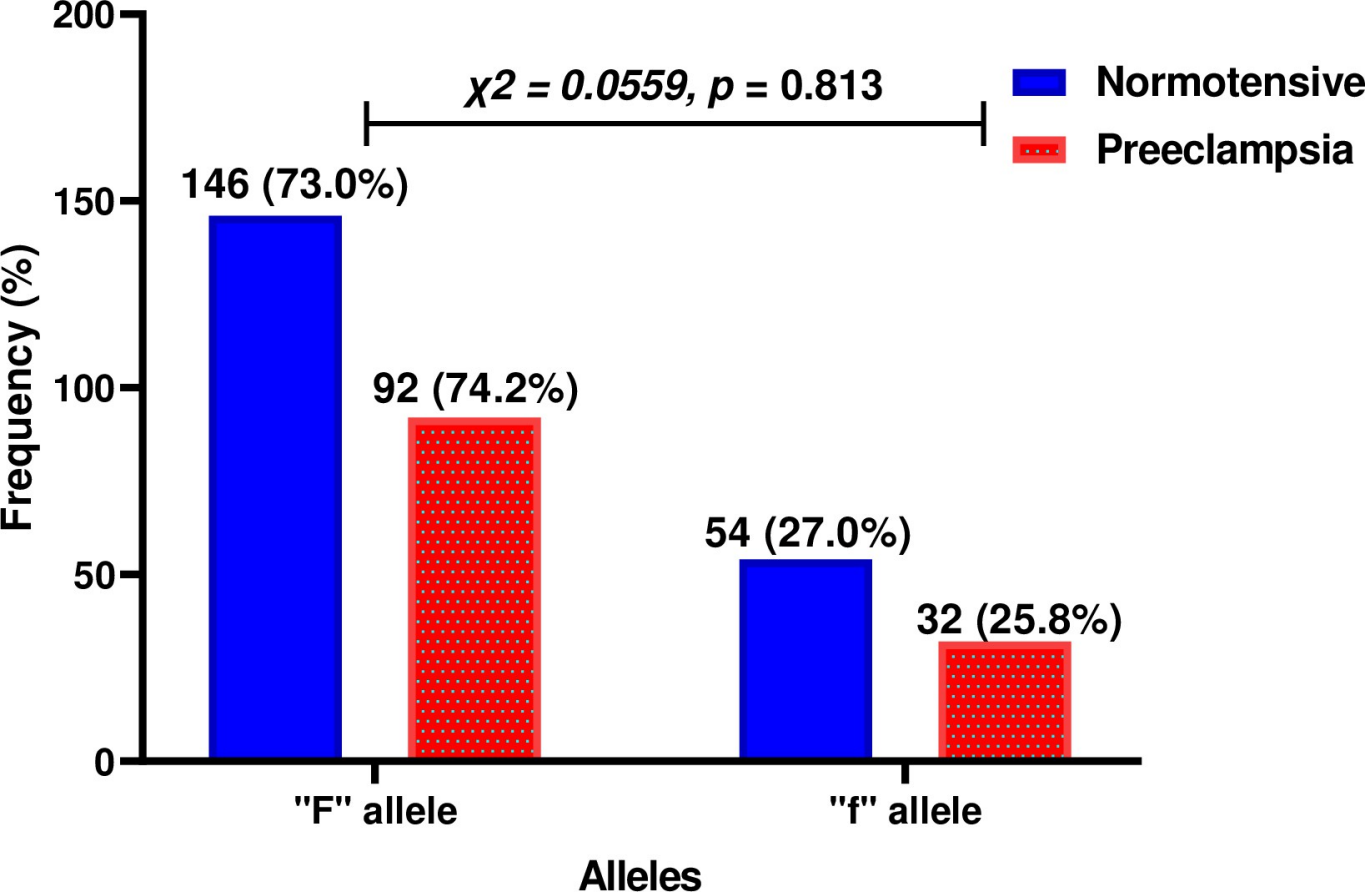
Allelic distribution of Fokl among study participants.

**Fig 4** below depicts the distribution of alleles of the Fok1 SNP, “F” and “f” among study participants, with 73% of the normotensive group and 74.2% of the preeclamptic group carrying “F” allele. Conversely, the “f” allele was present in 27% of the normotensive group and 25.8% of the preeclamptic group. There were no significant differences between the two groups in the distribution of both alleles (*p* = 0.813).

**Fig 5:**
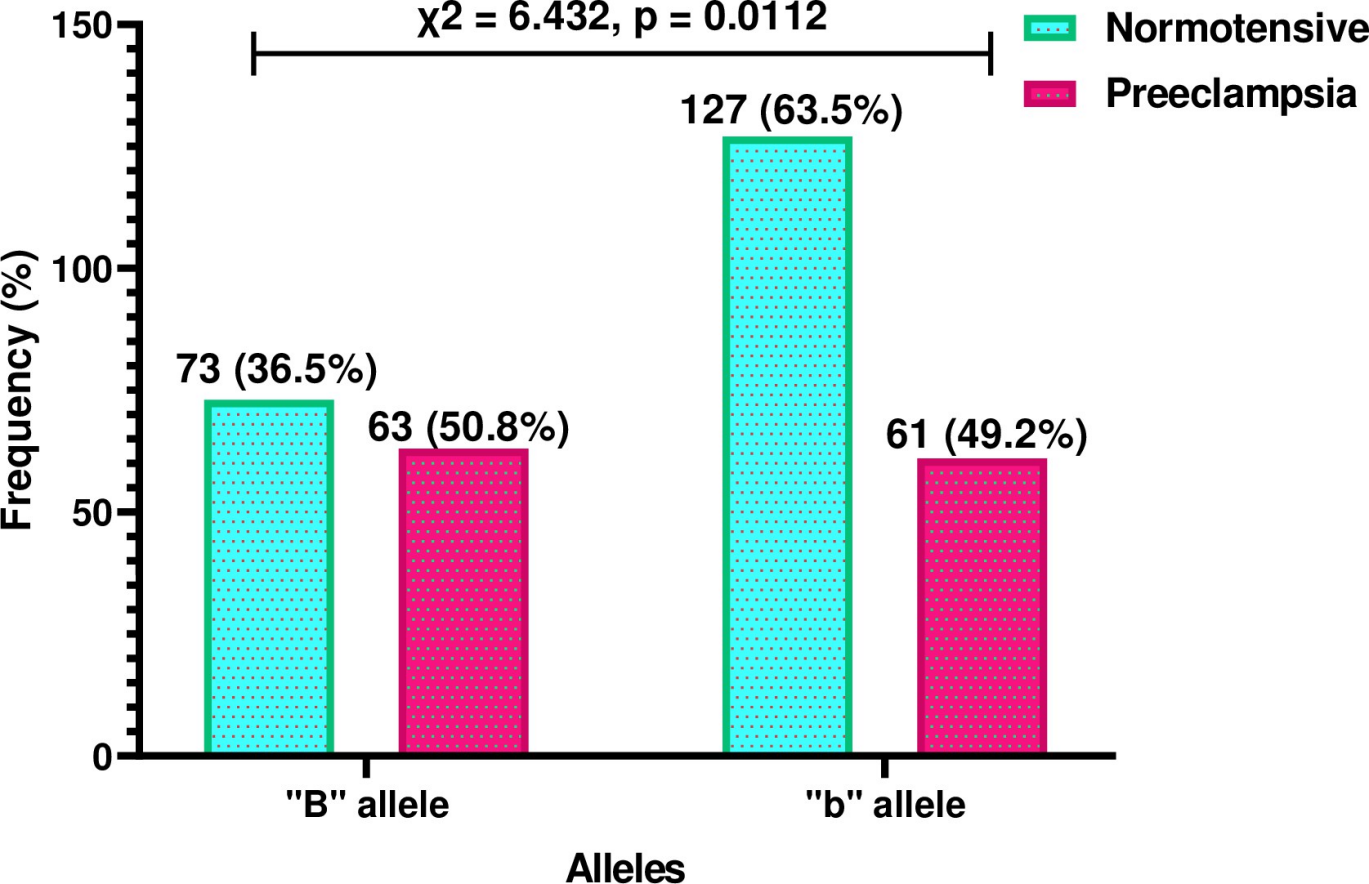
Allelic distribution of Bsm1 among study participants.

**Fig 5** shows the distribution of Bsml alleles, “B” and “b”, among the study participants. The “B” allele was found in 36.5% of the normotensive group and 50.8% of the preeclamptic group, while the “b” allele was observed in 63.5% of the normotensive group and 49.2% of the preeclamptic group. The distribution of these alleles showed statistical significance between the two groups (p = 0.0112).

## Discussion

This study investigated the relationship between vitamin D levels, VDR gene variants, and the risk of developing preeclampsia in Ghanaian women. This present study revealed a high prevalence of vitamin D deficiency (54.7%) among study participants. Notably, the concentration of 25(OH)D was significantly lower in the preeclampsia (PE) group compared to the normotensive group. Furthermore, vitamin D deficiency was significantly associated with a three-fold increased risk of PE, even after adjusting for age and BMI. In examining the role of specific VDR gene variants and PE risk, it was observed that individuals with the "bb" genotype (homozygous for the recessive Bsm1 allele) had a reduced risk of developing preeclampsia. While the genotypic distribution of Fok1 among both groups did not show any significant differences, there was a significant difference seen in the distribution of Bsm1. The distribution of alleles of Fok1 between the two groups did not show statistical significance while, the distribution of Bsm1 alleles between the two groups showed significant difference. The findings suggest a complex association between vitamin D, VDR gene variants, and preeclampsia among Ghanaian women.

Vitamin D plays a multifaceted role in pregnancy, including immune regulation, inflammation control, and influencing vascular and bone health [5]. The global challenge of vitamin D deficiency is well established, however it varies significantly across different populations and regions in the world [17]. This study found that despite Ghana’s tropical climate, the prevalence of vitamin D deficiency was high at 54.7%, which is consistent with previous studies in the Ghanaian population; a multi-center study by Sakyi *et al*., [18] reported a vitamin D deficiency prevalence of 43.6%. Likewise two separate studies b**y** Fondjo *et al*. [13, 19] reported a vitamin D deficiency prevalence of 81.7% among pregnant women and 92.2% among pre- and post-menopausal type II diabetes respectively. Although, the current prevalence is relatively lower compared to our earlier reported prevalence of 81.7% in pregnant women, the disparities could possibly be due to the 2 different study locations. Our earlier study was in the Volta region, southern part of Ghana whereas the current study was within the middle belt of Ghana with variations in ethnic groups and lifestyle factors. These findings however, highlight the extent of vitamin D deficiency in the Ghanaian population and suggest that relying on sunlight and diet for vitamin D synthesis may not be sufficient, particularly among pregnant women.

Remarkably, these findings align with the global trends of vitamin D deficiency prevalence that have been reported in other populations [5]. Other studies have however, reported contradictory findings to this phenomenon; a study in Sweden by Barenbring *et al*., [20] reported a lower prevalence of vitamin D deficiency of 10% in pregnant women, and 2% among women born in Northern Europe. A review by Spiro *et al*., [21] also reported vitamin D deficiency in European adults as ranging from 2% to 30% indicating the influence of factors like sun exposure patterns, seasons, cultural differences, and lifestyle practices on the variations of vitamin D deficiency observed in different places in the world [22].

Our findings also indicate a three-fold increase in the odds of developing preeclampsia among those with vitamin D deficiency, even after adjusting for age and BMI. These findings are consistent with our previous studies [13] that reported a significant difference in vitamin D concentration between preeclamptic and non-preeclamptic groups, and a four to five-fold increase in odds of developing preeclampsia for those with severe deficiency in a similar population. While some observational studies [23–25] also reported similar findings, others did not find any differences in vitamin D concentration between the two groups and there was no association between low maternal vitamin D levels and the risk of developing PE [26–28].

Disparities in findings among these studies can be attributed to different definitions of vitamin D deficiency cut-offs, seasonal variations, dietary habits, ethnicity, geographical locations, and the use of vitamin D supplements [11]. In Ghana, the practice of vitamin D supplementation for pregnant women is not common practice, as it has traditionally been largely assumed that sunlight is sufficient for vitamin D synthesis. The findings from this study challenges this assumption and emphasizes the need for public health policies addressing vitamin D deficiency in pregnancy. Some studies have suggested that vitamin D deficiency contributes significantly to the pathogenesis of preeclampsia due to its various roles in pregnancy, including immune regulation, placental implantation, vascular structure maintenance, and oxidative stress reduction [3]. Disruptions in these processes due to vitamin D deficiency could lead to abnormal placental implantation, vascular dysfunction and proteinuria.

Our present study showed that there were no significant differences in the frequencies of the Fok1 genotypes, between the cases and controls. The distribution of Bsm1 genotypes among the normotensive and PE groups also showed significant difference. However, being homozygous recessive “bb” for Bsm1 may confer a reduced risk for preeclampsia. It is likely the ‘bb’ genotype may modulate transcription and translation of the VDR protein leading to adequate vitamin D levels and ultimately reduce preeclampsia risk. A study by Rezavand *et al*., [10] in an Iranian population of Kurdish ethnic backgrounds, reported no association between Fok1, Bsm1, Taq1 VDR polymorphisms, or haplotypes and preeclampsia or gestational hypertension. Contrary to these findings, the VDR variant “Ff” was associated with a decreased risk of PE in a study by Farajian-mashadi et al., in Iranian population [6]. In this study involving Iranian pregnant women, Fok1 showed a decreased risk of PE, whereas no association was found between Taq1, Apa1, and Bsm1 with PE. Similarly, Zhan *et al*., [9] in a study conducted in a population of Chinese Han women found an association between Fok1, but not with Bsm1 and PE susceptibility. Wang *et al*., [29] in 2013 reported an association between the VDR Bsm1 and Fok1 polymorphisms with susceptibility to hypertension in a population of recruited participants in the United States of America. The variability in findings across different studies highlights the complexity of the genetic contributions to preeclampsia, influenced by population diversity, ethnic backgrounds, geographical locations and associated epigenetic influences [30]. It is necessary for additional studies to examine multiple SNPs as functional groups (haplotypes) within VDR variants to gain a more comprehensive understanding of their associations with diseases.

A much larger sample size might be required in future studies to augment associations, particularly VDR gene variant and preeclampsia in the Ghanaian population. Additionally, this was an observational study; this limit causal inferences, necessitating future longitudinal investigations. Furthermore, this study focused on a specific subset of VDR gene variants, and other polymorphisms that might contribute to preeclampsia susceptibility warrant exploration.

## Conclusion

There is a high prevalence of vitamin D deficiency among preeclamptic pregnant women. Vitamin D deficiency is associated with increased risk of preeclampsia. However, "bb" genotype of the VDR Bsm1 variant may decrease the risk for preeclampsia.

## Supporting information

S1 Data (XLSX)

S1 File (DOCX)
