# Supplementary figures and images for: Interplay between vitamin D status, vitamin D receptor gene variants and preeclampsia risk in Ghanaian women: A case-control study
Source: PLoS One. 2024 May 30;19(5):e0303778. doi: 10.1371/journal.pone.0303778 (PMC11139312; doi:10.1371/journal.pone.0303778)

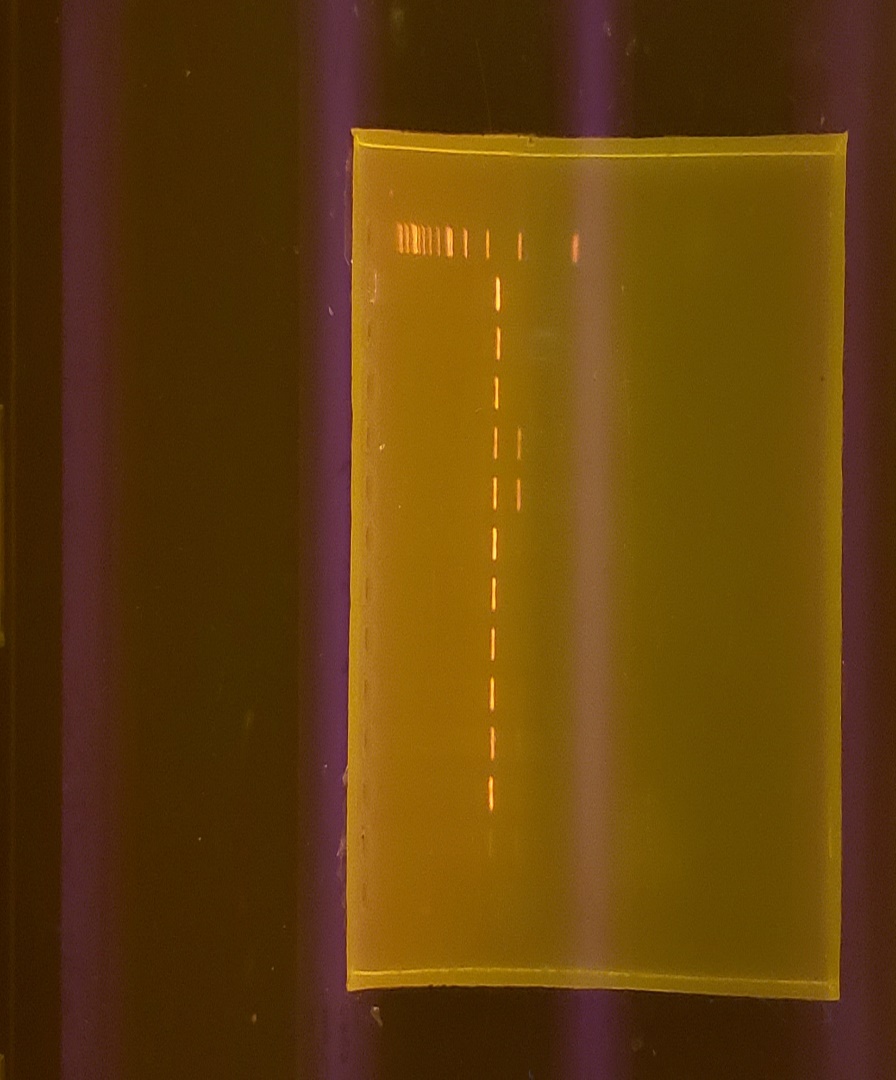


Raw unedited image for **Fok1 (Figure 1b)**


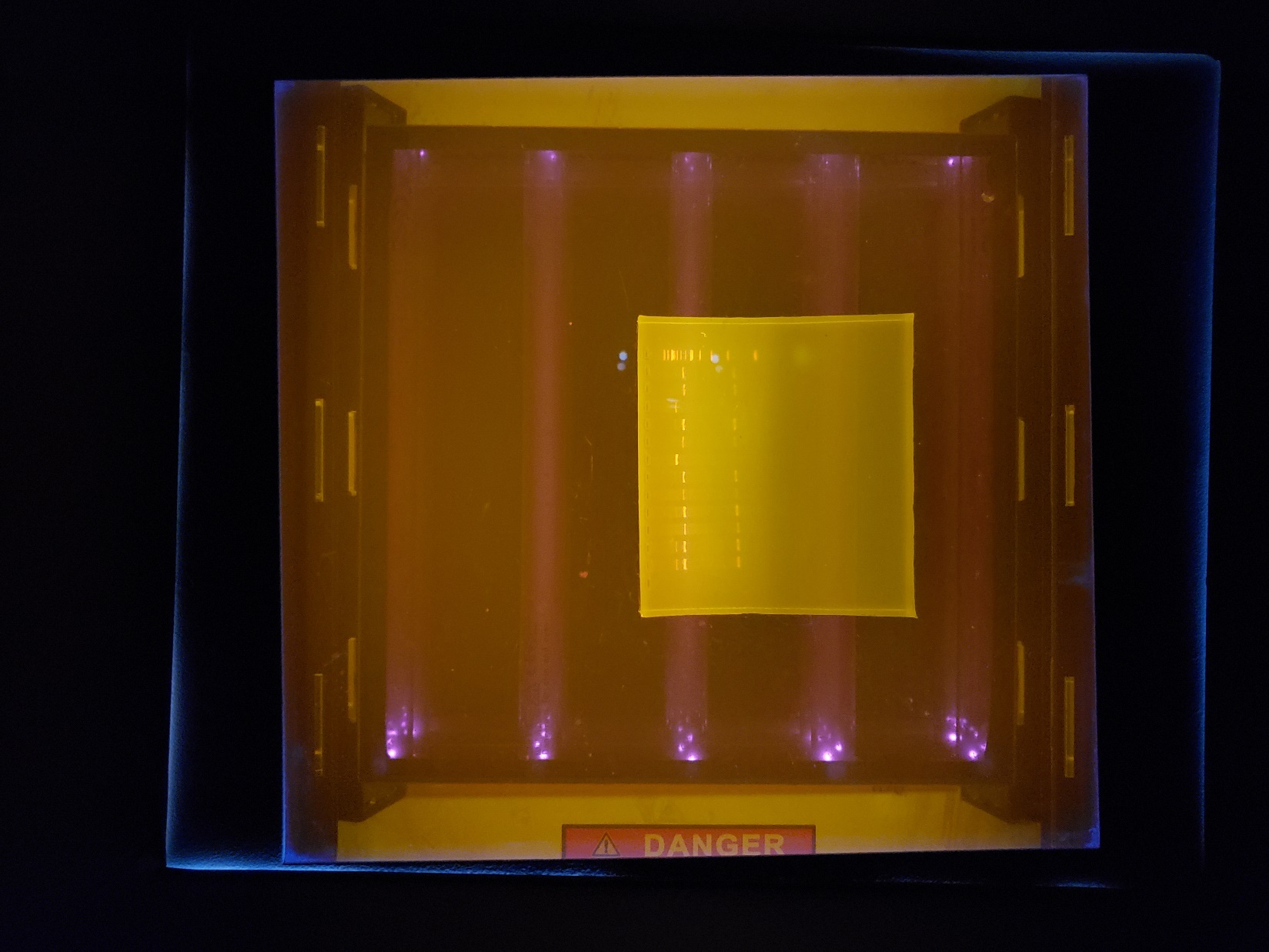


Raw unedited image for Bsm1 (**Figure 2b)**

Supplement: S1 File — (DOCX) [file pone.0303778.s002.docx]
